# Evaluating Social Protection Policies With an Implementation Science Framework: India’s Direct Benefit Transfer for Tuberculosis: Comment on "Does Direct Benefit Transfer Improve Outcomes Among People With Tuberculosis? – A Mixed-Methods Study on the Need for a Review of the Cash Transfer Policy in India"

**DOI:** 10.34172/ijhpm.2023.7698

**Published:** 2023-03-05

**Authors:** Ann M. Schraufnagel, Priya B. Shete

**Affiliations:** ^1^Division of Pulmonary and Critical Care Medicine, Zuckerberg San Francisco General Hospital, University of California, San Francisco, CA, USA; ^2^Center for Tuberculosis, University of California-San Francisco, San Francisco, CA, USA

**Keywords:** Tuberculosis, Cash Transfer, Social Protection, Implementation Science, India

## Abstract

Addressing the social and structural determinants of tuberculosis (TB) through social protection programs is a central feature of global public health policy and disease elimination strategies. However, how best to implement such programs remains unknown. India’s direct benefit transfer (DBT) program is the largest cash transfer program in the world dedicated to supporting individuals affected by TB. Despite several studies aimed at evaluating the impact of DBT, many questions remain about its implementation, mechanisms of action, and effectiveness. Dave and Rupani’s mixed-methods evaluation of this program previously published in this journal offers valuable insights into the strengths and limitations of the DBT program in improving TB treatment outcomes. Their results also provide an opportunity for demonstrating how systematically collected data may be further analyzed and presented using implementation science, a field of study using methods to promote the systematic uptake of evidence-based interventions to support sustainable program scale-up.

## Introduction

 Tuberculosis (TB) is a disease of poverty, disproportionately affecting the most socioeconomically vulnerable and precipitating increased impoverishment among those affected by the disease. A central feature in addressing this global public health challenge is integrating social protection interventions, strategies to reduce the social and financial burden of disease, into TB prevention and care programs. These interventions, which can include cash transfers, transportation vouchers, social health insurance, and disability benefits, among others, are included in the World Health Organization’s (WHO’s) End TB Strategy^1^ as well as the Sustainable Development Goals Agenda.^2^ Countries with a high burden of TB, many of which are considered low- or middle-income, are now tasked with designing and implementing such programs to support TB affected communities.

 As an example, India’s direct benefit transfer (DBT) program is the largest cash transfer program in the world dedicated to supporting individuals affected by TB.^3^ In the January 2022 edition of this journal, Dave and Rupani^4^ published their findings on the impact of the national DBT program, Nikshay Poshan Yojana, on TB treatment outcomes in Western India using a mixed methods approach. Launched in April 2018, this program provides INR 500 per month (~US$ 7) to each person receiving medication for TB treatment via a bank transfer. Described as “incentives for nutritional support to TB patients,”^3^ this scheme was designed to provide financial resources directly to patients to address the malnutrition that is a well-known barrier to effective TB treatment.^5^ Programs such as DBT may be considered social protections, defined as policies or programs designed to mitigate social or financial risk for vulnerable populations such as individuals with TB. Thus far, studies of India’s DBT program were performed in the immediate post-implementation period and found that it reached a limited portion of the population and suffered massive implementation challenges^6-8^ with variable impact on TB outcomes. One study from South India showed no association between receipt of DBT and TB treatment success, loss to follow-up, or death in the initial months of the DBT program,^9^ while other studies of DBT showed improved treatment outcomes in participants with TB who received cash transfers.^10^

 Dave and Rupani add quantitative evidence of the positive impact interventions such as DBT can have on improving TB treatment outcomes as well as qualitative insights on strengths and opportunities for programmatic improvement to implementation of the DBT program. By evaluating its effectiveness 18-24 months after the rollout of India’s DBT program, they were able to bypass many of the “growing pains” associated with deploying a new national policy. Their results suggest that well-implemented TB-specific cash transfers through the DBT program are feasible and are associated with better treatment outcomes; of 426 participants, 91% received DBT and 91% had successful treatment outcomes.

 The heterogeneity of results between this and prior studies is likely due in part to variability in implementation outcomes, such as reach and coverage of the cash transfer intervention, but was also exacerbated by the lack of a standardized analytic approach to program evaluation. The lack of a framework for synthesizing collected data limits the potential for producing generalizable knowledge to systematically improve program implementation. The importance of high-quality evaluation of the implementation and effectiveness of this DBT program cannot be underestimated. India accounted for 26% of global TB cases in 2020^5^ and DBT represents one of the only social protection programs for people with TB that has been implemented at-scale in a country with a high burden of TB. Lessons learned from this program could dramatically influence the implementation and scale-up of similar programs for at-risk individuals in places with high rates of TB, a priority for global TB elimination.^11^

## Can Tuberculosis Specific Cash Transfer Programs Be Effectively Implemented at Scale?

 Consistent with previous studies, Dave and Rupani found challenges to delivering DBT to include some participants’ — particularly marginalized populations’ — lack of bank accounts or possession of the documents required to open one, bureaucratic hurdles including delays and multiple checkpoints needed for delivery of DBT, and poor intervention uptake in the private sector.^4,6-8^ The effect of these implementation challenges on TB outcomes, however, is less clear. Dave and Rupani report that non-receipt of DBT (odds ratio [OR]: 5, confidence interval [CI]: 2-12) was significantly associated with unfavorable treatment outcomes, on par with the effect of being unemployed (OR: 4, CI: 2-10) or being HIV positive (OR: 6, CI: 2-23). However, late receipt of first and last installments of DBT were not associated with unfavorable treatment outcomes.^4^ These conflicting data call into question the mechanism by which the DBT cash transfer affected TB treatment adherence and clinical or public health outcomes.

 Cash transfers are hypothesized to work by mitigating the effect of disease and other shocks on households by facilitating access to care, offsetting costs associated with care, and reducing susceptibility to social determinants of health.^12^ DBT was designed to target malnutrition for people with TB as a major barrier to successful TB outcomes. Its design stems from the vast literature associating poor nutrition with TB incidence and adverse outcomes^5^ as well as the high prevalence of undernutrition in India.^13^ The mismatch between implementation outcomes and effectiveness outcomes described in this study highlight our lack of understanding of the mechanism by which DBT may work: while non-receipt of DBT was associated with unfavorable TB treatment outcomes, late receipt of DBT was not. This argues against improved nutritional status as the driver of better TB treatment outcomes as many participants received DBT after the completion of therapy. Furthermore, almost all study participants felt the amount of money received through DBT was insufficient to cover nutritional costs.^4^ Additional insights may be needed to understand the mechanism of action for DBT in order to optimize the effectiveness and scalability of this and similar programs.

## Implementation Science: Providing a Potential Framework for Social Protection Evaluation and Policy Analysis

 To organize and explore this further, we propose using an implementation science framework to evaluate this and other similar programs. Implementation science provides methodologies that promote the systematic uptake of evidence-based research into routine practice to improve the quality and effectiveness of health services by fostering an understanding of barriers, facilitators, and contextual factors and their mechanisms of action in intervention implementation.^11^ It considers individual, organizational, and societal factors in order to plan, influence, and evaluate interventions. We reframe results presented by Dave and Rupani using an implementation science approach to suggest how DBT implementation and evaluation may be more effective.

 The Consolidated Framework for Implementation Research (CFIR) is one well-known example of an implementation science framework which consolidates constructs found in a broad array of behavioral theories to provide a structure for analysis of intervention implementation across many settings.^12^ With five main domains (innovation characteristics, outer setting, inner setting, characteristics of individuals, and process), it can be used to generate an action-oriented theory of change and evaluate an existing intervention. Once barriers to implementation have been identified, an associated tool, the Expert Recommendations for Implementing Change (ERIC),^15^ can be used to help choose validated implementation strategies to mitigate barriers. The value of this approach is in using validated tools to map programmatic performance gaps to barriers and facilitators central to implementation, and then further to identify concrete strategies that may overcome those challenges.

 In Figure, we present a reorganization of the themes identified in Dave and Rupani’s analysis into CFIR domains (“Determinants”). Each of the themes could be categorized according to specific determinants within the implementation ecosystem. These determinants include those that reflect the larger policy context and broad implementation environment (“outer setting”) as well as those most proximate to implementation including the hyperlocal environment in which implementation occurs (“inner setting”), the “characteristics of the individuals” participating, and the “process.” Using the CFIR-ERIC tool, we mapped these determinants to implementation strategies, several of which were supported by suggestions from program participants and functionaries as noted in Dave and Rupani’s supplementary data. Replicating this exercise could help stakeholders prioritize revisions to the DBT strategy based on feasibility and impact, targeting the most influential factors in the DBT implementation ecosystem.

**Figure:**
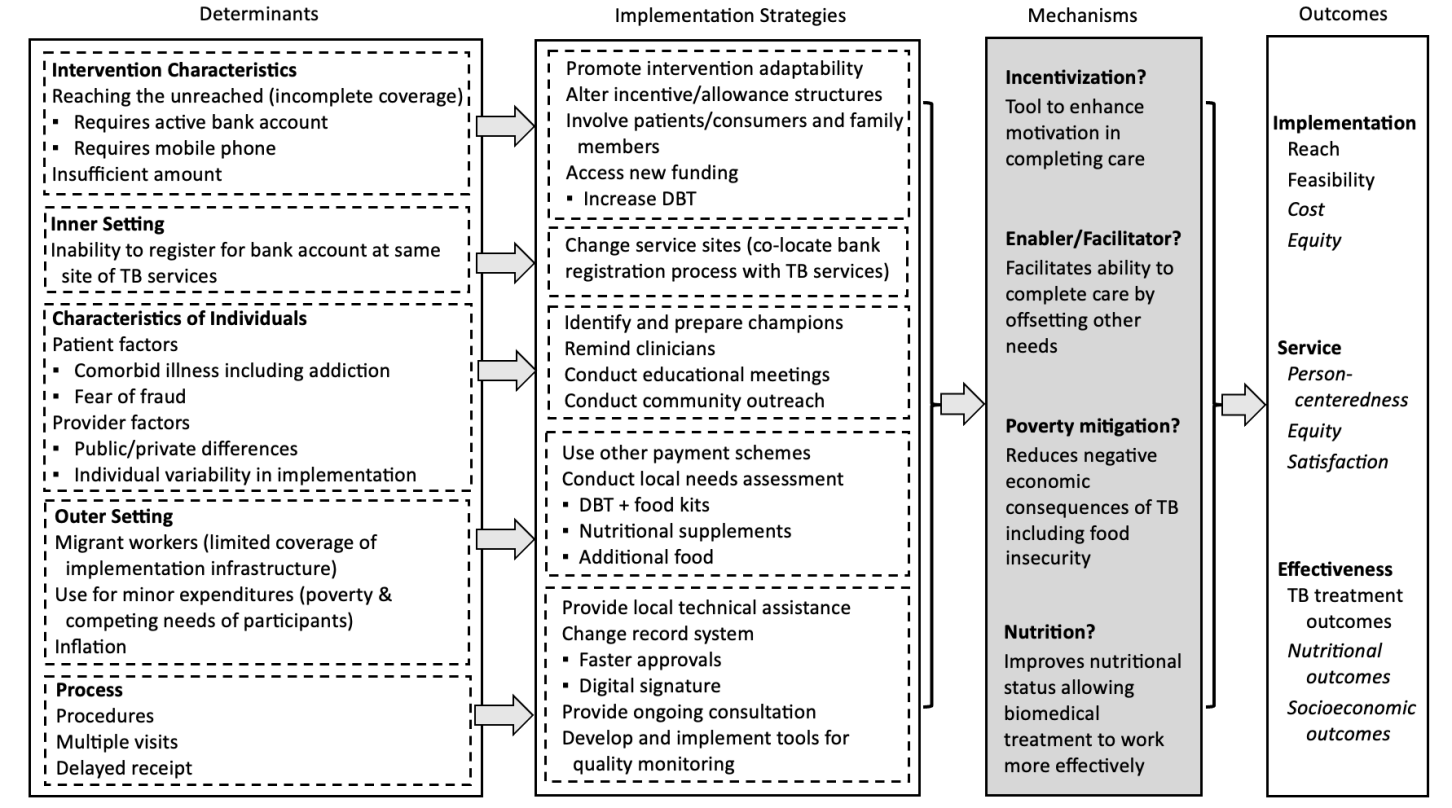
Mapping the determinants of DBT success to potential implementation strategies using CFIR-ERIC. The five CFIR domains include (1) Intervention characteristics (characteristics of the intervention being implemented into a particular organization); (2) Inner setting (structural, political, and cultural contexts through which the implementation process proceeds); (3) Characteristics of individuals (individuals involved with the intervention and/or implementation process, including consideration of their cultural, professional, and individual mindsets and affiliations); (4) Outer setting (economic, political, and social context within which an organization resides); and (5) Process (process of implementation, aimed to achieve individual and organizational level use of the intervention as designed).^15^ We used data presented by the authors to systematically map determinants – factors reported as barriers or facilitators of DBT – to validated implementation strategies noted in the CFIR-ERIC tool (with sub-headings reflecting specific results noted in Dave and Rupani’s qualitative analysis). We highlight in grey where our knowledge gaps remain. Currently unexplored person-centered outcomes are presented in italics. Abbreviations: DBT, direct benefit transfer; CFIR, Consolidated Framework for Implementation Research; ERIC, Expert Recommendations for Implementing Change; TB, tuberculosis.

 Presenting data collected by Dave and Rupani in this format also highlights gaps in our knowledge and opportunities for future research. The mechanisms of action for DBT and other similar social protections are often unknown, and yet optimizing their effectiveness and scalability demands understanding how they work. To generate this evidence, intermediate implementation outcomes should be evaluated and their effect on TB outcomes explored. For example, quantifying the proportion of recipients who purchased food with their cash transfers as well as changes in weight or body mass index could validate DBT’s function as a nutritional support. If on the other hand we hypothesize that DBT works by motivating adherence, investigating client perceptions and behavioral effects of the intervention would be necessary. Finally, if these cash transfers prevent further impoverishment in people with TB, measuring socioeconomic indicators in addition to food security would help explain if DBT functions as a social protection. Further, these mechanisms of action may differ for different populations, in different contexts, and foster other important outcomes related to equity, client satisfaction, improved quality of care, or socioeconomic impacts. Systematically reviewing data through an implementation science lens can elaborate mechanisms of action and and identify potential benefits essential to person-centered care, even beyond TB outcomes.

## Conclusion

 Given the strong association between nutrition, poverty, and TB, the WHO’s End TB Strategy^1^ highlights social support, nutritional support, and social protection as integral to eradicating TB and improving patient outcomes. In this high incidence TB^5^ setting, India’s well-implemented government social protection program for TB affected individuals could be a model for how countries can effectively target social and structural determinants of disease, making a real impact on the global TB epidemic. Using implementation science frameworks to evaluate and modify the DBT intervention has the potential to both optimize the impact of DBT while also creating more generalizable evidence of how programs like this may be implemented and scaled to address this global health priority.

## Ethical issues

 Not applicable.

## Competing interests

 Authors declare that they have no competing interests.

## Authors’ contributions

 AMS and PBS equally contributed to conception or design of the commentary, as well as interpretation of data; drafting and revising the manuscript and provided final approval of the version to be published.

## Funding

 AMS’s time was supported by a NIH/NHLBI T32 award.
